# Key Factors and Parameter Ranges for Immune Control of Equine Infectious Anemia Virus Infection

**DOI:** 10.3390/v15030691

**Published:** 2023-03-06

**Authors:** Dylan Hull-Nye, Tyler Meadows, Stacey R. Smith?, Elissa J. Schwartz

**Affiliations:** 1 Department of Mathematics, Washington State University, Pullman, WA 99164, USA; 2 Department of Mathematics and Statistics, Queen’s University, Kingston, ON K7L 3N6, Canada; 3 Department of Mathematics, Faculty of Medicine, The University of Ottawa, Ottawa, ON K1N 6N5, Canada; 4 Department of Mathematics and Statistics, School of Biological Sciences, Washington State University, Pullman, WA 99164, USA

**Keywords:** Equine Infectious Anemia Virus, cytotoxic T lymphocytes, antibodies, bifurcations, Latin hypercube sampling, partial rank correlation coefficients, least squares

## Abstract

Equine Infectious Anemia Virus (EIAV) is an important infection in equids, and its similarity to HIV creates hope for a potential vaccine. We analyze a within-host model of EIAV infection with antibody and cytotoxic T lymphocyte (CTL) responses. In this model, the stability of the biologically relevant endemic equilibrium, characterized by the coexistence of long-term antibody and CTL levels, relies upon a balance between CTL and antibody growth rates, which is needed to ensure persistent CTL levels. We determine the model parameter ranges at which CTL and antibody proliferation rates are simultaneously most influential in leading the system towards coexistence and can be used to derive a mathematical relationship between CTL and antibody production rates to explore the bifurcation curve that leads to coexistence. We employ Latin hypercube sampling and least squares to find the parameter ranges that equally divide the endemic and boundary equilibria. We then examine this relationship numerically via a local sensitivity analysis of the parameters. Our analysis is consistent with previous results showing that an intervention (such as a vaccine) intended to control a persistent viral infection with both immune responses should moderate the antibody response to allow for stimulation of the CTL response. Finally, we show that the CTL production rate can entirely determine the long-term outcome, regardless of the effect of other parameters, and we provide the conditions for this result in terms of the identified ranges for all model parameters.

## 1. Introduction

Equine Infectious Anemia Virus (EIAV) is a lentivirus that infects horses, ponies, donkeys and other equids. EIAV is transmitted between hosts by biting flies [1] and is more prevalent in warmer climates [2]. Only 11 equid infections require reporting to the OIE, the World Organization for Animal Health, of which EIAV is one [3]. The virus is similar in genetic makeup, structure, genome and life-cycle to Human Immunodeficiency Virus (HIV) [4]. However, EIAV can be controlled by the equine immune system—jointly by cytotoxic T lymphocytes (CTLs) and antibodies—leading to a persistent but asymptomatic infection. In contrast, HIV is not generally controlled by the human immune system and can eventually develop into acquired immune deficiency syndrome (AIDS). As such, EIAV is valuable for research focused on the development of prophylactic vaccines against EIAV and related lentiviruses, including HIV [5,6,7].

There is no treatment for EIAV infection, which is typically characterized by three stages: the acute stage has high fever and low platelet count; the chronic stage has spiking viral load, wasting and recurring febrile episodes; and the asymptomatic stage has decreased viral load and an absence of clinical symptoms [4]. Prevention is usually managed by quarantine or euthanasia of animals that test positive for infection [8]. No vaccinations are currently used, although control of EIAV in China between 1975 and 1990 was attributed to a live-attenuated vaccine used during a pilot program [8,9]. Nevertheless, the existence of severe combined immunodeficiency (SCID) horses—horses without an adaptive immune system—makes EIAV an ideal case study for investigating the immune response. Studies of EIAV infection in SCID horses have added to the understanding of how both antibody responses and CTLs control infection [10,11]. A successful vaccine for HIV infection, therefore, will ideally stimulate both the CTL response and the antibody response, as has frequently been suggested [12,13].

Several prophylactic vaccines have been tested for EIAV that used inactivated whole virus or recombinant envelope subunit vaccine approaches [14,15,16], although none have been released, because the studies yielded variable results [8]: some vaccines protected the horses from infection, while others increased viral replication. A live-attenuated vaccine approach is believed to promise the greatest efficacy, but it is thought to be too risky due to the potential for a lentivirus to revert to higher virulence. Thus, in this work, we use a mathematical modeling approach to investigate immune-system components that correlate with protection. Importantly, the immune correlates of EIAV protection from disease or infection are still unknown [17].

Previous modeling of EIAV analyzed virus and infected-cell dynamics with two viral strains and constant or decaying antibody levels [18], examined the protective effect of antibodies against multiple mutants [19], calculated quantitative kinetic estimates of antibody escape [20], showed that long-term dynamics depend on the ratio of cell-to-cell versus free-virus transmission [21] and calculated key kinetic parameters in the absence of adaptive immunity [22]. Other studies used EIAV as a case study to examine the mechanics of viral infection without cytopathicity [23], applied homotopy analysis to determine semi-analytical solutions [24], utilized global stability to estimate EIAV parameters [25], showed the existence of a Hopf bifurcation in a model of EIAV infection with a delay in the CTL response [26], and determined the number of vaccinations needed to prevent mutant escape [27].

This work expands upon an existing model of EIAV infection [28,29]. The model has three steady states: an infection-free equilibrium, a boundary equilibrium (containing antibodies but no CTLs) and a coexistence equilibrium (containing both antibodies and CTLs). Initial research determined the closed form of all steady states, the basic reproductive number $R_{0}$, a threshold $R_{1}$ that delineates two of the steady states [28] and conditions for the global stability of the infection-free equilibrium and local stability of the boundary and endemic equilibria [29]. A subsequent analysis showed that the system could undergo two transcritical bifurcations, depending on the CTL proliferation rate ψ and the antibody production rate α. These parameters represent the strength of the adaptive immune response and are viable targets for a potential vaccine. A two-parameter bifurcation diagram constructed using these parameters showed two stable regions: one for the boundary and one for the endemic equilibrium [29].

Here, we used the slope of the slant asymptote to a degenerate hyperbola in order to determine the focused ranges of Latin hypercube sampling (LHS) parameters that lead to heightened sensitivity near the bifurcation boundary. This allowed for us to investigate the following research questions: (1) Which parameter regimes lead to stability of the coexistence equilibrium? (2) What levels of antibody or CTL production would provide optimal stimulation of the immune response against viral infection? Using LHS and partial rank correlation coefficients (PRCCs), we assessed the parameter sensitivity around a threshold above which the coexistence equilibrium is stable. We then identified which parameters and parameter ranges guarantee particular system outcomes, thereby determining potential mechanisms of focus for vaccine development.

## 2. The Model

The model [28] consists of a system of five ordinary differential equations (ODEs) describing changes in the number of healthy macrophages *M*, virus particles *V*, infected macrophages *I*, CTLs *C* and antibody particles *A*:

(1a) $$\begin{array}{cc} \frac{dM}{dt} & =\lambda -\rho M-\beta MV, \end{array}$$

(1b) $$\begin{array}{cc} \frac{dI}{dt} & =\beta MV-\delta I-kIC, \end{array}$$

(1c) $$\begin{array}{cc} \frac{dV}{dt} & =bI-\gamma V-fVA, \end{array}$$

(1d) $$\begin{array}{cc} \frac{dC}{dt} & =\psi IC-\omega C, \end{array}$$

(1e) $$\begin{array}{cc} \frac{dA}{dt} & =\alpha V-\mu A. \end{array}$$

A full description of the parameters and their units can be found in Table 1.

The initial parameter ranges in this study were based upon both known and unknown values. Estimates for the parameters ρ, β, δ, λ, *b* and γ were determined in a previous study [22] by data fitting to SCID horses with EIAV infection; in the current study, the initial parameter ranges (Table 1, unshaded columns) were chosen broadly around the interquartile ranges of those estimates [22]. The parameters *f*, *k*, ψ, ω, α and μ are unknown; hence, initial ranges (unshaded columns) were drawn from simulations that produced the full display of model dynamics, as in previous mathematical studies [28,29]. Within these initial ranges, a sample value was found in the vicinity of one of the system’s bifurcation curves, and the ranges were then revised (shaded columns) using a 15% band on either side of these sample values, as described below.

This system has three biologically relevant equilibria: an infection-free equilibrium $E_{0}$, in which the only non-zero state variable is *M*; a boundary (or antibody-only) equilibrium $E_{1},$ in which all state variables except for *C* are positive and $C^{*}=0$; and a coexistence equilibrium $E_{3},$ in which all state variables are positive. There are also two non-biologically relevant equilibria, $E_{2}$ and $E_{4}$, that always have at least one negative component.

The infection-free equilibrium $E_{0}$ is globally asymptotically stable when the basic reproductive number

(2) $$R_{0}=\frac{\beta \lambda b}{\delta \gamma \rho }$$

is less than one. When $R_{0}=1$, the system undergoes a transcritical bifurcation. When $R_{0}>1$, the infection-free equilibrium $E_{0}$ is unstable. The system undergoes a second transcritical bifurcation when

(3) $$R_{1}=\frac{\hat{V}\beta \lambda \psi }{\delta \omega \left(\rho +\beta \hat{V}\right)}=1,$$

where

(4) $$\hat{V}=\frac{-\gamma \mu \psi +\sqrt{{\left(\gamma \mu \psi \right)}^{2}+4\psi f\alpha b\omega \mu }}{2\psi f\alpha }.$$

When $R_{1}<1<R_{0}$, the boundary equilibrium $E_{1}$ is locally asymptotically stable; when $1<R_{1}<R_{0}$, the coexistence equilibrium is locally asymptotically stable. A summary of the conditions for existence and stability of the three equilibria is given in Table 2. More information about the exact forms of equilibria can be found in [28,29].

## 3. The Boundary and Coexistence Regions

Since EIAV is a persistent infection, the virus is retained at low levels indefinitely (for the lifetime of the infected host). Thus, we only observe either the $E_{1}$ or $E_{3}$ state in infected horses, so our investigations focus on what distinguishes the coexistence region (when $E_{3}$ is stable) from the boundary region (when $E_{1}$ is stable). In the coexistence region, the joint adaptive immune response controls the virus, which is maintained at a low but nonzero level.

**Figure 1:**
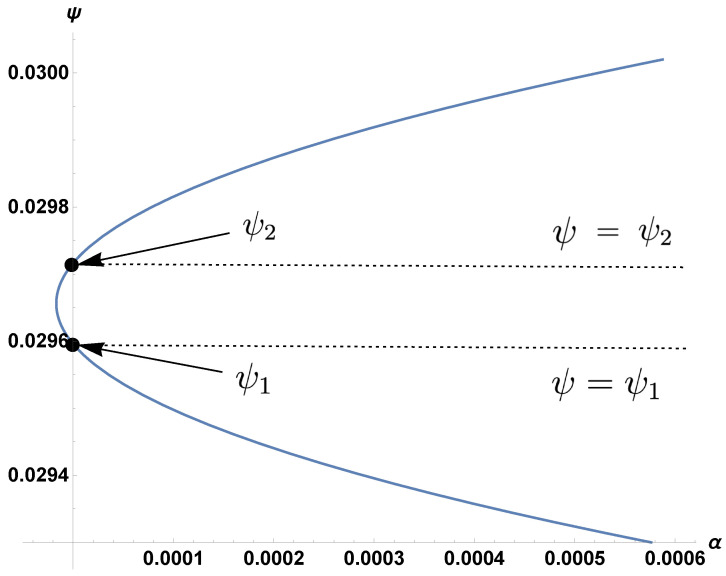
Hyperbola crossing the ψ-axis with horizontal lines $\psi =\psi_{1}$ and $\psi =\psi_{2}$ using “sample value” parameters from the “revised” column of Table 1. Note the relatively small parameter ranges of α and ψ.

**Figure 2:**
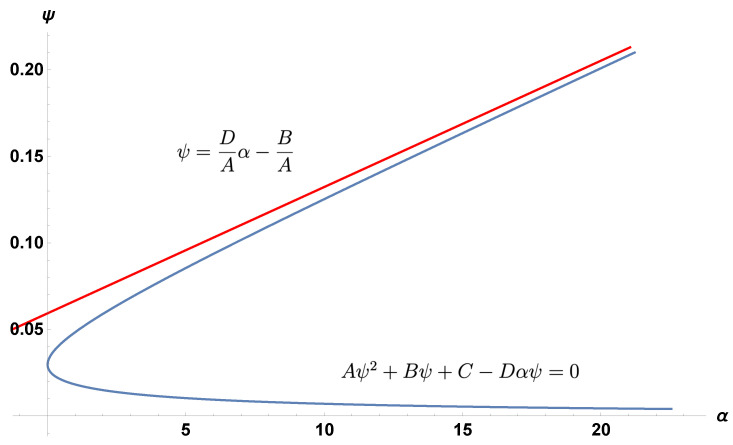
The slant asymptote (in red) and the degenerate hyperbola (in blue), using parameter values set to “sample value” from the “revised” column of Table 1.

In Equations (1d) and (1e), the CTL and antibody proliferation rates are ψ and α. In Meadows and Schwartz [29], numerical plots of the α–ψ relationship were generated for a specific set of parameter values. A quasi-linear curve divides the region diagonally from the origin (with ψ on the vertical axis and α on the horizontal axis). The upper region corresponds to the stability of the coexistence equilibrium, and the lower region corresponded to the stability of the boundary region. Here, we show that the curve is a degenerate hyperbola [30] and, for any choice of positive parameter values, the hyperbola always produces a positively sloped slant asymptote; see Figure 1 and Figure 2. Therefore, an inverse relationship always exists between ψ and α, i.e., higher values of ψ or lower values of α lead the system towards the coexistence of CTLs and antibodies. The mathematical results are summarized as follows.

**Lemma** **1.** *In the α–ψ plane, the curve separating the coexistence region from the boundary region is a degenerate hyperbola of the form $A\psi^{2}+B\psi +C-D\alpha \psi =0$ where A,C,D>0 and B<0.*

**Proof.**  The curve separating the two regions is defined by the transcritical bifurcation that occurs when stability transfers from $E_{1}$ to $E_{3}$. Therefore, it can be implicitly represented by the equality $R_{1}=1$. Note that by Theorem 3 of [28], $R_{0}>R_{1}$, and thus $R_{0}>1$ along this curve. Expanding $R_{1}$ and $\hat{V}$, we can obtain

(5) $$\beta \left(\lambda \psi -\delta \omega \right)\sqrt{{\left(\gamma \mu \psi \right)}^{2}+4\psi f\alpha b\omega \mu }=2\rho \psi f\alpha \delta \omega +\left(\lambda \psi -\delta \omega \right)\left(\beta \psi \mu \gamma \right).$$

Squaring both sides and rearranging leads to the hyperbola

(6) $$\begin{array}{c} A\psi^{2}+B\psi +C-D\alpha \psi =0, \end{array}$$

where

(7a) $$\begin{array}{cc} A & =b\beta^{2}\lambda^{2}\mu \omega -\beta \delta \gamma \lambda \mu \omega \rho , \end{array}$$

(7b) $$\begin{array}{cc} B & =\beta \delta^{2}\gamma \mu \omega^{2}\rho -2b\beta^{2}\delta \lambda \mu \omega^{2}, \end{array}$$

(7c) $$\begin{array}{cc} C & =b\beta^{2}\delta^{2}\mu \omega^{3}, \end{array}$$

(7d) $$\begin{array}{cc} D & =\delta^{2}f\omega^{2}\rho^{2}. \end{array}$$

This hyperbola is degenerate since

$$\det\left[\begin{array}{cc} 0 & -\frac{D}{2}\\ -\frac{D}{2} & A \end{array}\right]=-\frac{D^{2}}{4}<0.$$

Since all parameters are positive, C>0 and D>0. By factoring, we can rewrite *A* as

$$\begin{array}{c} A=\left(\beta \lambda \mu \omega \right)\left(\delta \gamma \rho \right)\left(R_{0}-1\right), \end{array}$$

which is positive since $R_{0}>R_{1}=1$. Similarly, *B* can be factored as

$$\begin{array}{c} \begin{array}{c} B=\frac{\beta \mu \omega^{2}\delta }{\delta \gamma \rho }\left(1-2R_{0}\right), \end{array} \end{array}$$

which is negative since $R_{0}>R_{1}=1$.

In order to investigate how the hyperbola changes as the parameters vary, we examined the intersection between the curve and the ψ-axis (α=0). Due to the nature of the threshold equations (2) and (3), the curve always intersects the ψ-axis at two distinct points, creating three regions: upper, middle, and lower; see Figure 1. However, coexistence only applies to the intersection of the upper region and the portion of the interior above $\psi =\psi_{1}$.

**Proposition** **1.** *The coexistence region corresponds to the area above the hyperbolic curve; the boundary region corresponds to the area below the curve. The slope of the slant asymptote is $\frac{D}{A}$, with intercept $-\frac{B}{A}$.*

**Proof.**  On the ψ-axis, we have the two roots

(8) $$\begin{array}{cc} \psi_{1} & =\frac{\delta \omega }{\lambda }, \end{array}$$

(9) $$\begin{array}{cc} \psi_{2} & =\frac{b\beta \delta \omega }{b\beta \lambda -\delta \gamma \rho }. \end{array}$$

These roots have the relationship $\psi_{2}=\left(\frac{R_{0}}{R_{0}-1}\right)\psi_{1}$, which implies $\psi_{2}>\psi_{1}$ because $R_{0}>1$. From Equation (5), it can also easily be shown that

(10) $$\begin{array}{c} \psi \lambda -\delta \omega >0 \end{array}$$

in the coexistence region (see Appendix A).

In other words, the coexistence equilibrium $E_{3}$ is only stable in the region $R_{1}>1$ (the area outside the hyperbola) and above the line $\psi =\psi_{1}$. The intersection of these two regions results in only the upper portion of the hyperbola and the ψ-axis. When $R_{1}<1$, the boundary equilibrium $E_{1}$ is stable, mapping to the region inside the hyperbola.

Finally, the equations of the asymptotes can be found by setting C=0 in Equation (6) and factoring into the two lines

(11) $$\begin{array}{cc} \psi & =0, \end{array}$$

(12) $$\begin{array}{cc} \psi & =\frac{D}{A}\alpha -\frac{B}{A}. \end{array}$$

Equation (12) is the slant asymptote.

**Figure 3:**
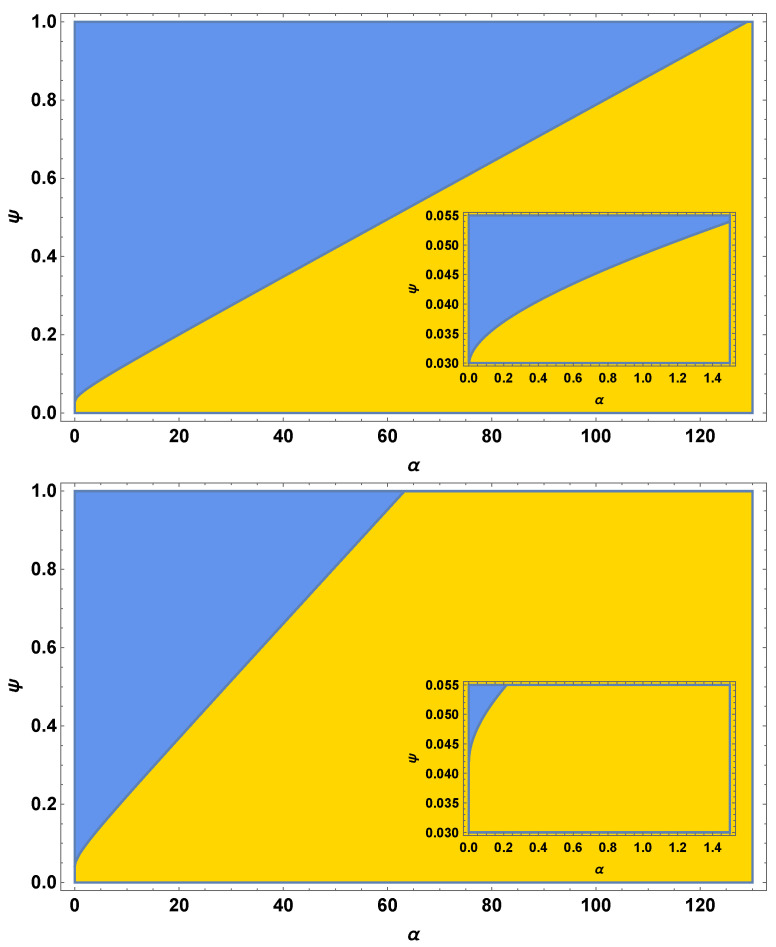
The top figure shows the degenerate hyperbola using “sample value” parameters from Table 1, with λ = 31. The bottom figure uses the same values but with λ = 22. The blue shaded region (top left of each graph) corresponds to stability of the coexistence equilibrium $E_{3}$; the yellow shaded region (bottom right of each graph) corresponds to stability of the boundary equilibrium $E_{1}$. The insets illustrate the curvature of the hyperbola near the origin.

In Figure 3, we show how the hyperbola splits the positive quadrant into two regions. In the region above the hyperbola, the coexistence equilibrium $E_{3}$ is stable. In the region below the upper branch of the hyperbola, the boundary equilibrium $E_{1}$ is stable. Figure 3 also demonstrates how the parameters affect the slope of the hyperbola. Decreasing λ decreases the value of *A*, which increases the slope of the slant asymptote and decreases the relative size of the coexistence region.

## 4. Parameter Ranges and Sensitivity Analysis

### 4.1. Parameter Ranges

In order to determine critical parameter ranges for the degenerate hyperbola, we first set the initial ranges (for parameters other than ψ and α) to the min and max values in Table 1. These were then used to find revised ranges that generated the separated regions seen in [29]. To generate these ranges, we searched parameter space via a Latin hypercube sample of size *n* = 10,000 drawn from uniform distributions between the min and max values in Table 1.

The slope of the asymptote of the degenerate hyperbola satisfies

$$\frac{\psi_{max}-\psi_{min}}{\alpha_{max}-\alpha_{min}}.$$

In this example, since ψ ranges from 0 to 1 and α ranges from 0 to 150, the slope that splits the regions into approximately equal parts is $\frac{1}{150}$. To determine parameters producing the slope nearest this value, we calculated $\frac{D}{A}$ for each draw of the Latin hypercube sample and then use the least-squares method [31,32] to minimize the distance to $\frac{1}{150}$. The revised values are shown in Table 1 (shaded columns), along with a lower bound 15% below the sample value and an upper bound 15% above the sample value. Note that the sample values are not the medians of min and max, nor is it expected that the median for every parameter would necessarily produce the critical slope.

**Figure 4:**
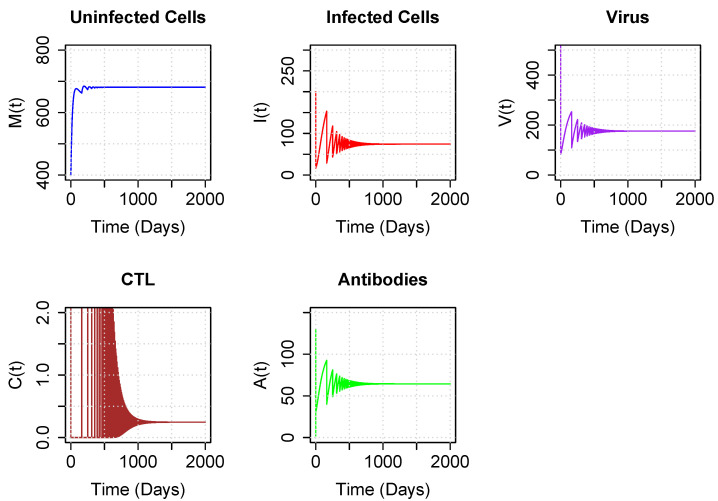
Trajectory plots of populations for parameters corresponding to the coexistence region, above the asymptote. Parameter values are set to sample values of Table 1, with ψ=0.7 and α=20. Initial conditions are $M_{0}=400$, $I_{0}=200$, $V_{0}=10,000$, $C_{0}=10$ and $A_{0}=2$.

Using our revised parameter values for model parameters other than ψ and α, we next numerically demonstrated that the system converges to either the coexistence equilibrium or the boundary equilibrium depending only on the values of ψ and α. Figure 4 illustrates the long-term dynamics of the model using parameters for ψ and α drawn from the coexistence region above the asymptote. Note that both CTLs and antibodies persist after 2000 days, reaching an equilibrium, following damped oscillations. Interestingly, studies of experimental infections of EIAV show oscillating kinetics in viral load [33,34,35], as well as in neutralizing antibodies and Env-specific CTLs [17], before reaching equilibrium.

**Figure 5:**
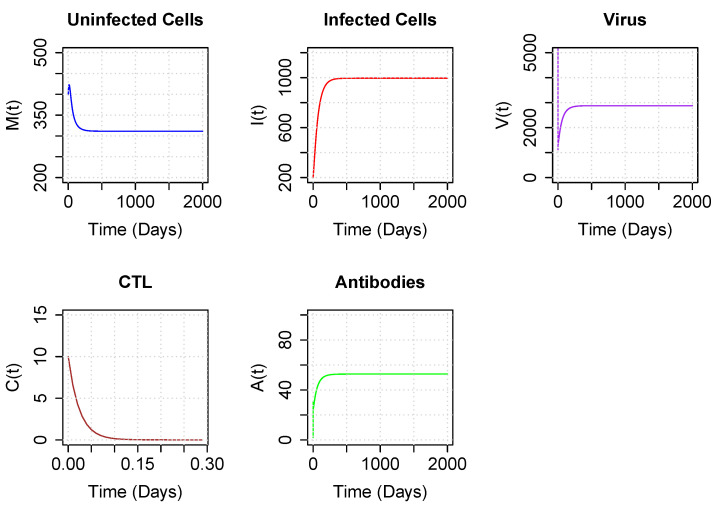
Trajectory plots of populations for parameters corresponding to the coexistence region below the asymptote but above the upper branch of the hyperbola. Parameter values are set to sample values of Table 1, with ψ=0.05 and α=1. Initial conditions are $M_{0}=400$, $I_{0}=200$, $V_{0}$ = 10,000, $C_{0}=10$ and $A_{0}=2$. Note the very brief time scale for C(t).

Figure 5 illustrates the long-term dynamics of the model using parameters for ψ and α drawn from the coexistence region below the asymptote but above the upper branch of the hyperbola. In this case, CTLs quickly decline, while antibodies persist at moderate levels. Although CTL levels appear close to zero, they are positive.

**Figure 6:**
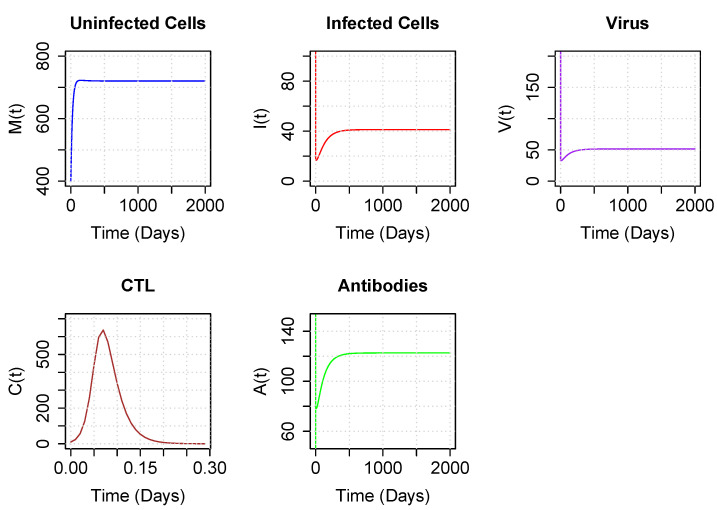
Trajectory plots of populations for parameters corresponding to the boundary region below the upper branch of the hyperbola. Parameter values are set to sample values of Table 1, with ψ=0.7 and α=130. Initial conditions are $M_{0}=400$, $I_{0}=200$, $V_{0}=10,000$, $C_{0}=10$ and $A_{0}=2$. Note the very brief time scale for C(t) quickly converging to 0 (under one day).

Finally, Figure 6 illustrates the long-term dynamics of the model using parameters for ψ and α drawn from the boundary region. In this case, the CTLs go to zero, while the antibodies are maintained at a high level.

### 4.2. Sensitivity Analysis

In order to examine the sensitivity of parameters on the steady state, we used LHS to sample parameter space and PRCCs for the measure of correlation between each parameter and an output. LHS samples parameters from a grid without row or column replacement, while PRCCs rank the effect of varying each parameter against the median of the other parameters, regardless of whether that effect is positive or negative [36].

Our output was $R_{1}$, since this is the threshold for the coexistence equilibrium. The input was a set of n=10,000 random LHS draws of parameter space, selected from a uniform distribution. The method [36] calculates PRCC values by establishing two linear regression models: one between each input parameter and the other parameters; the second between the output variable and the other parameters. After ranking all variables, the PRCC is the Spearman correlation coefficient between the residuals of these two models. The LHS-PRCC method has been shown to work well in many contexts, including for global sensitivity analysis [37] and especially for non-linear output variables in deterministic models [38].

**Figure 7:**
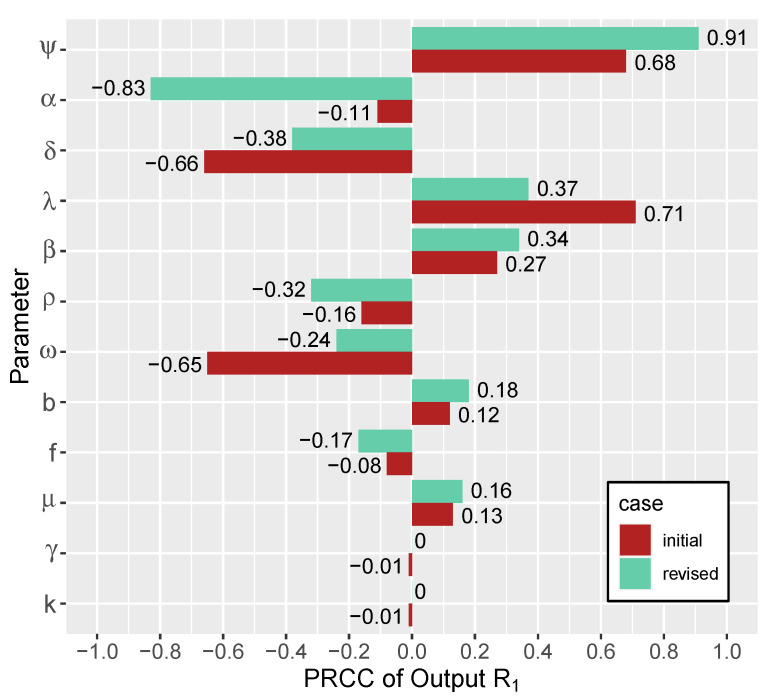
Tornado plot of the PRCC values for each parameter for initial min and max ranges (red/bottom bars for each parameter) and revised lower and upper ranges (green/top bars for each parameter) using values from Table 1. Note the increased trends from ψ and α using the revised ranges, whereas δ, λ and ω lose significance close to the hyperbola.

The LHS sample for our sensitivity analysis was based on the initial ranges and the revised ranges (±15% ranges around the sample values) from Table 1. In Figure 7, PRCCs using the initial ranges (bottom bars for each parameter, shown in red) suggest that λ,ψ,δ and ω are the most significant parameters. For example, if the infected cells or CTLs are dying off slowly (i.e., if δ or ω take on small values), the system moves towards coexistence, since these parameters are strongly negatively correlated with $R_{1}$; if the infected cells or CTLs die too quickly (i.e., if δ or ω are large), the system moves towards the boundary region. The initial ranges had high PRCCs for ψ but low PRCCs for α (Figure 7). Using the revised ranges (top bars for each parameter, shown in green), however, ψ and α became the most significant parameters, while δ, λ and ω lost significance near the hyperbola. With the revised ranges, we observed that greater values of ψ move the system towards coexistence, while greater values of α move it towards the boundary equilibrium. In other words, higher production rates of CTLs, along with lower production rates of antibodies, cause the system to tend towards simultaneous antibody and CTL responses, whereas the boundary region is more likely to be stable when there are higher antibody production rates and lower CTL production rates.

**Figure 8:**
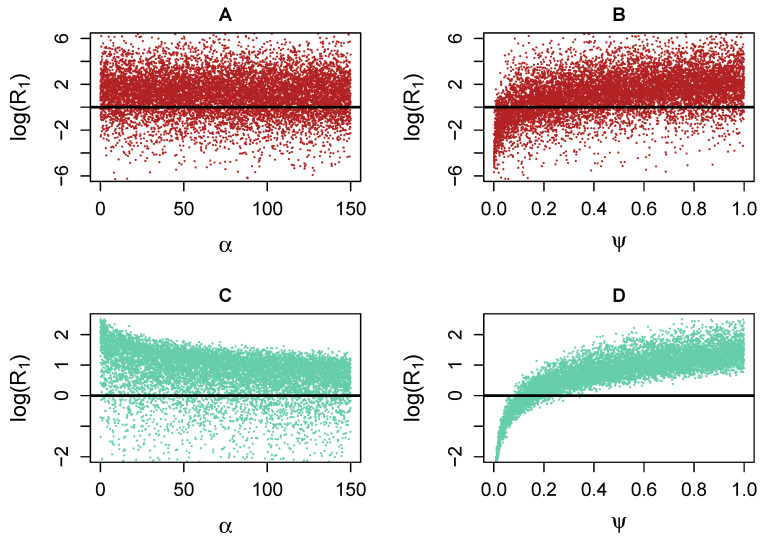
Scatter plots associated with Figure 7 using LHS-sampled data (each dot is a simulation). Top row: initial case. Bottom row: revised case. Note the different vertical scales in each plot. The threshold $R_{1}=1$ is depicted with a horizontal line. (**A**): approximately uniform distribution of α in the initial ranges. (**B**): increasing trend in ψ in the initial trend, but no range exists wholly below the threshold. Note the distinct trend captured for ψ in the lower right figure (**D**): sufficiently small values of ψ correspond to the case of no coexistence, regardless of the values of other parameters. Conversely, sufficiently large values of ψ will guarantee coexistence. The same properties do not apply to α (**C**).

Next, we depicted the sensitivity of the $R_{1}$ threshold to parameters ψ and α using landscape scatter plots. The values of ψ and α were varied over their initial or revised ranges, holding all other parameters at the median values of their ranges. Figure 8 demonstrates that no clear correlations are seen using the initial ranges (i.e., neither ψ nor α lead to coexistence or CTL eradication), though increasing values of ψ trend slightly towards coexistence (i.e., $R_{1}>1$). However, with the revised ranges, ψ alone can determine coexistence outcomes (for ψ>0.3) or antibody-only outcomes (for ψ<0.1), regardless of other parameters. Conversely, there is no such pattern for α, despite the decreasing trend in $R_{1}$ as α increases.

The reason that ψ can control the outcome on its own is that increasing ψ from any point inside the boundary region of Figure 1 will always lead to the coexistence region. Conversely, decreasing α horizontally from some points inside the boundary region leads to coexistence, but decreasing α horizontally from other points does not: namely, those starting below the $\psi =\psi_{2}$ line.

Note that the PRCC is a Spearman correlation (ranked), which is a measure of monotonicity between two variables. It is also useful to see a non-ranked correlation, such as Pearson, which measures the regression coefficient to determine the degree of linear correlation: for α, it is r=−0.53, and for ψ, it is r=0.57, both significantly lower than than their PRCC values. This is likely due to the nonlinearity in the relationship with $R_{1}$.

## 5. Discussion

EIAV is a persistent infection in which the viral load is not cleared but is retained indefinitely. Thus, long-term dynamics will not reach an infection-free equilibrium; instead, they will be in the form of either an antibody equilibrium in the absence of CTLs (i.e., antibody-only) or an equilibrium where antibodies and CTLs coexist. Here we determined the threshold conditions that delineate coexistence from the antibody-only state in order to highlight the effect of parameter changes on the long-term outcome. Key parameters whose values can determine the outcome provide insight into successful vaccine development by identifying which vaccine characteristics to target.

Our analyses showed that boosting CTL production rates (ψ) and moderating antibody growth rates (α) both favour coexistence provided the model parameters are in specified ranges, as shown in the “revised” columns of Table 1. In order to remain in the coexistence region, high ψ values and low α values are required, although the region extends the allowable α values if ψ is large. Our results support the conclusions of earlier work [28,29], but we additionally identified a set of candidate parameter ranges. Furthermore, we showed that the CTL production rate ψ can entirely determine the long-term outcome, regardless of the effect of other parameters, for ranges near the threshold. Specifically, values of ψ greater than 0.3 will lead to the coexistence of antibodies and CTLs, regardless of the other parameter values, while values of ψ below 0.1 will lead the system to an immune response in which only the antibody response is sustained (the antibody-only equilibrium).

Our model has some limitations, which should be acknowledged. Our model does not include all parts of the immune system in detail, including B cell dynamics. Other authors have included more model complexity, such as by including B cell dynamics and germinal center generation ([39]), but in our case, this approach would require additional parameters with unknown values for EIAV infection. Our model uses the straightforward approach in which antibody production is proportional to the viral concentration. This choice still depicts the immunological mechanism at play, given that antibody production is correlated with the virus quantity [35,40,41].

Both neutralizing and non-neutralizing antibodies are seen in EIAV infection [42]. Our model assumes that the antibodies block virus particles, forming an antibody–virus complex that is removed from the system, as in previous modeling by other authors [43]. Since this blocking reduces virus (*V*), it also affects the βMV infection term, although indirectly. Increasing the killing rate of infected cells via antibody-dependent cell cytotoxicity (ADCC) was not included in the model because the control of EIAV has been shown to not be dependent on ADCC-mediating antibodies [44].

While it is difficult to ascertain how realistic the unknown parameters are, the revised ranges (Table 1, shaded values) offer theoretical guidance on how the parameter values correspond to model outcomes. In terms of parameter sensitivity, the tornado plot shows that the model is more sensitive to λ, δ, ω and ψ with the initial ranges, and less sensitive—relatively—to λ, δ and ω with the revised ranges. In the future, more models are needed that consider the time course of immune responses to evaluate the stages of infection at which each immune response is most relevant.

Although the usual metrics in vaccine trials are antibody levels or virus-specific CTL levels, our study suggests that assessing the CTL and antibody production rates will provide an indication of whether a potential vaccine can give rise to a low-level, asymptomatic, persistent infection. When measuring CTL levels and antibody levels, the production rates of CTLs and antibodies are implied, since boosting the levels with vaccination would result from production. Taken a step further, our results would recommend that experiments be developed that can measure CTL production rates and antibody production rates or, even more pertinently, the ratio of CTL to antibody production. A CTL production exceeding antibody production on the order of 150:1 would predict a low-level, asymptomatic, persistent infection.

In general, our method could be applied to other models in which an explicit asymptote can be derived, or for other ranges, other pairs of parameters or other bifurcation curves. Here, the bifurcation curve always delineates two regions: an upper coexistence region and a lower boundary region, varying only via the angle made by the slant asymptote. This asymptote depends on all parameters except *k*, the rate at which CTLs kill infected cells.

Although most HIV infections are not controlled by the immune system, some are. The existence of long-term non-progressors with low or undetectable viral loads [45] suggests that there may be some key parallels between the immune control of HIV and EIAV. A few mathematical and computational modeling studies have addressed HIV sequence diversity correlations with immune control [46], and the role of set point viral load (SPVL) in HIV transmissibility [47]. Future studies are warranted, however, which incorporate SPVL into mathematical models of lentiviral immune control. Such models are needed to examine the mediators of and connection between SPVL and immune system correlates of HIV long-term non-progression. Other authors have likewise called for such future studies [48,49].

Our results have applications to HIV vaccine development. Vaccine researchers have debated whether an HIV vaccine should focus on stimulating T cell responses or antibody responses. The earliest trials primarily focused on the production of neutralizing antibodies, but, due to disappointing results, attention turned to developing vaccines to stimulate the T cell response. When a trial showed efficacy associated with the production of functional binding antibodies, the focus in the field returned to stimulating broadly neutralizing antibody production [50]. Mounting evidence from untreated infected individuals who control their infections, however, lends support to the development of HIV vaccines that stimulate T cells in conjunction with those that stimulate antibody responses [46].

We note that EIAV-infected equids with only an antibody response have not been witnessed; clinical and experimental EIAV-infected equids with long-term asymptomatic infections show that CTLs and antibodies are required to control the viral load and clinical disease [8,10]. However, our simulated trajectories show the antibody-only state displayed a lower viral load, a lower infected cell count, a higher uninfected cell count and higher antibody levels than the coexistence state. While the antibody-only state may control the virus, the fact that it has not been observed suggests that it is not a viable path to immune control. Thus, it is likely that coexistence (i.e., persistent CTL and antibody levels) is the only realistic possibility that accurately reflects immune system control of persistent viral infection, consistent with recommendations regarding the optimal vaccine strategy for HIV infection [12,13,46]. To reach coexistence, the parameter ranges offered in this study provide minimal and maximal values that can serve as a guide in vaccine development.

## Figures and Tables

**Table 1:** Parameter definitions and ranges. The initial range expresses a naïve parameter set, which was then used to find sample values in the vicinity of the hyperbola asymptote using 10,000 Latin hypercube samples. The revised range was then determined by focusing on a 15% band above and below the sample values.

Definition	Units	Initial		Revised		
		Min	Max	Lower	Sample Value	Upper
Uninfected arrival (λ)	$\frac{cells}{mL\times day}$	0	50	26.35	31	35.65
Uninfected death (ρ)	$\frac{cells}{day}$	0	0.05	0.036	0.042	0.048
Infectivity rate (β)	$\frac{mL}{vRNA\times day}$	$3\times 10^{-7}$	$10^{-3}$	$1.7\times 10^{-5}$	$2\times 10^{-5}$	$2.3\times 10^{-5}$
Infected cell death (δ)	$\frac{cells}{day}$	0	0.05	0.015	0.018	0.021
Killing by CTLs (*k*)	$\frac{mL}{cell\times day}$	$10^{-4}$	0.1	0.049	0.058	0.067
Virus production (*b*)	$\frac{vRNA}{cell\times day}$	100	10000	2006	2360	2714
Virus clearance (γ)	$\frac{cells}{day}$	0	20	7.5	8.82	10.14
Ab neutralization (*f*)	$\frac{mL}{molec\times day}$	0	30	13.03	15.33	17.63
CTL production (ψ)	$\frac{mL}{cell\times day}$	0	1	0	0.5	1
CTL death (ω)	$\frac{cells}{day}$	0	200	44.19	51.98	59.78
Ab growth (α)	$\frac{molec}{vRNA\times day}$	0	150	0	75	150
Ab clearance (μ)	$\frac{Antibodies}{day}$	0	200	46.42	54.62	62.81

**Table 2:** Steady states of the model. Equilibria $E_{2}$ and $E_{4}$ are never biologically relevant, so they are omitted. Thresholds and steady states were determined in [28,29].

Steady-State	Stability of Steady-State	Interpretation	Form
$E_{0}$	$R_{1}<R_{0}<1$	Infection-Free	$\left(M^{*},0,0,0,0\right)$
$E_{1}$	$R_{1}<1<R_{0}$	Boundary	$\left(\bar{M},\bar{I},\bar{V},0,\bar{A}\right)$
$E_{3}$	$1<R_{1}<R_{0}$	Coexistence	$\left(\hat{M},\hat{I},\hat{V},\hat{C},\hat{A}\right)$

## Data Availability

No new data were created.

## Appendix A. Derivation of Inequality (uid15)

We begin with $R_{1}>1$. It follows that

(A1) $$\begin{array}{c} \frac{{\hat{V}}_{1}\beta \lambda \psi }{\delta \omega \left(\rho +\beta {\hat{V}}_{1}\right)}>1\\ {\hat{V}}_{1}\beta \lambda \psi >\delta \omega \left(\rho +\beta {\hat{V}}_{1}\right)\\ \left(\frac{-\gamma \mu \psi +\sqrt{{\left(\gamma \mu \psi \right)}^{2}+4\psi f\alpha b\omega \mu }}{2\psi f\alpha }\right)\beta \lambda \psi >\delta \omega \left(\rho +\beta \left(\frac{-\gamma \mu \psi +\sqrt{{\left(\gamma \mu \psi \right)}^{2}+4\psi f\alpha b\omega \mu }}{2\psi f\alpha }\right)\right)\\ \left(-\gamma \mu \psi +\sqrt{{\left(\gamma \mu \psi \right)}^{2}+4\psi f\alpha b\omega \mu }\right)\beta \lambda \psi >\delta \omega \left(2\rho \psi f\alpha +\beta \left(-\gamma \mu \psi +\sqrt{{\left(\gamma \mu \psi \right)}^{2}+4\psi f\alpha b\omega \mu }\right)\right)\\ -\gamma \mu \psi \beta \lambda \psi +\beta \lambda \psi \sqrt{{\left(\gamma \mu \psi \right)}^{2}+4\psi f\alpha b\omega \mu }>2\rho \psi f\alpha \delta \omega -\gamma \mu \psi \beta \delta \omega +\beta \delta \omega \sqrt{{\left(\gamma \mu \psi \right)}^{2}+4\psi f\alpha b\omega \mu }\\ \beta \lambda \psi \sqrt{{\left(\gamma \mu \psi \right)}^{2}+4\psi f\alpha b\omega \mu }-\beta \delta \omega \sqrt{{\left(\gamma \mu \psi \right)}^{2}+4\psi f\alpha b\omega \mu }>2\rho \psi f\alpha \delta \omega -\gamma \mu \psi \beta \delta \omega +\gamma \mu \psi \beta \lambda \psi \\ \beta \left(\lambda \psi -\delta \omega \right)\sqrt{{\left(\gamma \mu \psi \right)}^{2}+4\psi f\alpha b\omega \mu }>2\rho \psi f\alpha \delta \omega +\gamma \mu \psi \beta \left(\lambda \psi -\delta \omega \right) \end{array}$$

In order to derive a contradiction, suppose λψ−δω<0. Then, from inequality (A1), we have

$$\begin{array}{c} \beta \left(\lambda \psi -\delta \omega \right)\sqrt{{\left(\gamma \mu \psi \right)}^{2}+4\psi f\alpha b\omega \mu }-\gamma \mu \psi \beta \left(\lambda \psi -\delta \omega \right)>2\rho \psi f\alpha \delta \omega \\ \left(\lambda \psi -\delta \omega \right)\left(\sqrt{{\left(\gamma \mu \psi \right)}^{2}+4\psi f\alpha b\omega \mu }-\gamma \mu \psi \right)>\frac{2\rho \psi f\alpha \delta \omega }{\beta }>0 \end{array}$$

Hence, we have

$$\begin{array}{c} \left(\lambda \psi -\delta \omega \right)\left(\sqrt{{\left(\gamma \mu \psi \right)}^{2}+4\psi f\alpha b\omega \mu }-\gamma \mu \psi \right)>0 \end{array}$$

This leaves the possibility of only $\sqrt{{\left(\gamma \mu \psi \right)}^{2}+4\psi f\alpha b\omega \mu }<\gamma \mu \psi$. Upon squaring both sides and re-arranging, we have 4ψfαbωμ<0, which is a contradiction since all parameters are positive. Note that if λψ−δω=0, then we would have 0>2ρfαδω, which is also a contradiction. Therefore, we must have λψ−δω>0.
